# Mechanisms involved in nicotinamide adenine dinucleotide phosphate (NADPH) oxidase (Nox)-derived reactive oxygen species (ROS) modulation of muscle function in human and dog bladders

**DOI:** 10.1371/journal.pone.0287212

**Published:** 2023-06-23

**Authors:** Nagat Frara, Dania Giaddui, Alan S. Braverman, Kais Jawawdeh, Changhao Wu, Michael R. Ruggieri, Sr, Mary F. Barbe

**Affiliations:** 1 Center for Translational Medicine at the Lewis Katz School of Medicine, Temple University, Philadelphia, Pennsylvania, United States of America; 2 Department of Biochemistry and Physiology, Faculty of Health and Medical Sciences, University of Surrey, Guildford, United Kingdom; 3 Department of Neurosurgery, Massachusetts General Hospital, Boston, Massachusetts, United States of America; Max Delbruck Centrum fur Molekulare Medizin Berlin Buch, GERMANY

## Abstract

Roles of redox signaling in bladder function is still under investigation. We explored the physiological role of reactive oxygen species (ROS) and nicotinamide adenine dinucleotide phosphate (NADPH) oxidase (Nox) in regulating bladder function in humans and dogs. Mucosa-denuded bladder smooth muscle strips obtained from 7 human organ donors and 4 normal dogs were mounted in muscle baths, and trains of electrical field stimulation (EFS) applied for 20 minutes at 90-second intervals. Subsets of strips were incubated with hydrogen peroxide (H_2_O_2_), angiotensin II (Ang II; Nox activator), apocynin (inhibitor of Noxs and ROS scavenger), or ZD7155 (specific inhibitor of angiotensin type 1 (AT1) receptor) for 20 minutes in continued EFS trains. Subsets treated with inhibitors were then treated with H_2_O_2_ or Ang II. In human and dog bladders, the ROS, H_2_O_2_ (100μM), caused contractions and enhanced EFS-induced contractions. Apocynin (100μM) attenuated EFS-induced strip contractions in both species; subsequent treatment with H_2_O_2_ restored strip activity. In human bladders, Ang II (1μM) did not enhance EFS-induced contractions yet caused direct strip contractions. In dog bladders, Ang II enhanced both EFS-induced and direct contractions. Ang II also partially restored EFS-induced contractions attenuated by prior apocynin treatment. In both species, treatment with ZD7155 (10μM) inhibited EFS-induced activity; subsequent treatment with Ang II did not restore strip activity. Collectively, these data provide evidence that ROS can modulate bladder function without exogenous stimuli. Since inflammation is associated with oxidative damage, the effects of Ang II on bladder smooth muscle function may have pathologic implications.

## Introduction

Bladder pathology is associated with bladder muscle dysfunction. It has been documented that oxidative stress is closely associated with pathological mechanisms and symptoms of urinary bladder dysfunction [1–11]. For example, hydrogen peroxide (H_2_O_2_) has been shown to be associated with bladder pathophysiology in different species [12–15]. Yet, the physiological role of reactive oxygen species signaling in normal bladder function is still under investigation.

Reactive oxygen species (ROS) are chemically reactive oxygen-containing molecules that are generated during normal metabolic activity inside the cell, and have been found to be fundamental signaling molecules involved in many pathophysiological processes [16]. Among the ROS, only H_2_O_2_ is considered as long-lived. After being generated, it has the ability to cross cell membranes to reach distant sites, causing cellular and intracellular damage and eventually oxidative stress either solely or after it is converted to other shorter-living ROS [17]. Application of H_2_O_2_ to muscles causes an elevation of intracellular calcium ions, increase in muscle tone, and enhancement of electrical field stimulation (EFS) induced contractions [1, 18–22]. Also, H_2_O_2_ has been shown to play a role in mediating intracellular signaling pathways of many pathophysiological responses [23].

The NADPH oxidase (Nox) enzyme has been found to exist in almost every cell type [24]. It generates excessive superoxide in response to many pathological stimuli, causing oxidative damage. Although ROS can be generated by different enzymes in the body, Nox enzymes are the only enzymes shown to produce ROS as their sole function [25] and thus offer specificity over any other ROS-generating enzymes in the body. Under physiological conditions, Nox enzymes generate a low level of superoxide that are primarily involved in redox signaling required for normal organ function, although excessive Nox-derived superoxide production can damage tissues and organs [26].

Although oxidative stress has been directly linked to urinary bladder pathologies [1–4, 6–11, 27], the role of Nox-derived ROS in normal bladder function is poorly understood. For example, it has been demonstrated recently that Nox is the main source of ROS overproduction observed in a mouse bladder with cyclophosphamide induced cystitis [28]. However, the relationship between ROS regulation and Nox activity and normal bladder physiology has not been fully elucidated. Understanding such mechanisms in normal bladders is warranted for better knowledge of potential treatment strategies for patients with urinary dysfunctions resulting from increased oxidative stress, such as those caused by ischemia/reperfusion injury [29].

Therefore, the aim of this study was to use *in vitro* muscle strip contractility studies to explore the roles of ROS and Nox in regulating muscle function, and to examine mechanisms of Nox activation, in normal bladders from humans and dogs. We used human tissues to show the relevance of Nox and ROS regulation of bladder function to human health. Dogs were chosen to inform research on bladder dysfunction in dogs, and because they have physiological similarities to humans not present in other species and are suitable for certain clinical measurements, neurophysiological studies, and pharmacological investigations.

## Materials and methods

### Tissue procurement

A total of 7 human bladders (5 males and 2 females) were procured as whole human bladders from human organ transplant donors from the National Disease Research Institute (Philadelphia, PA). Donor age ranged between 22 and 57 years, with an average age of 46 ± 11.4 years. Of the 6 human donners, 5 were White, 1 was Black, and 1 was Hispanic. Those 7 bladders were transferred to us through the procurement agency as de-identified tissues. Since these human tissues samples were de-identified and were not used in clinical investigations, their use did not require Institutional Review Board approval under the common Rule of Protection of Human Subjects Regulation. That said, their use was approved by the Temple University Institutional Biosafety Committee (# 10799) and met Biosafety in Microbial and Biosafety Laboratory and OSHSA Standards.

This study also utilized a total of 4 normal control dogs, 3 males and 1 female. The 3 males were mixed-breed hound dogs, 6–8 months old, weighing 20–25 kg (Marshall BioResources, North Rose, NY). The female dog was an adult beagle, 8 months old, that was obtained from Envigo Global Services, Inc. Denver, PA. All experiments performed on dog tissues were approved by the Institutional Animal Care and Use Committee according to guidelines of the National Institute of Health for the Care and Use Laboratory Animals and the United States Department of Agriculture and the Association for Assessment and Accreditation of Laboratory Animal Care. Dogs were group housed according to the institution’s standard husbandry with 12-hr exposure to light/dark cycles. The male dogs were sham-operated control animals derived from other larger studies focusing on nerve transfer for pelvic organ reinnervation or heart failure.

### Bladder muscle strip contractility studies

Each of the whole human bladders collected from 7 organ transplant donors were used for the *in vitro* muscle strip contractility studies. The specimens were harvested within 30 min after cross-clamping the aorta and transported to the laboratory within 40 hours immersed in Belzer’s Viaspan® University of Wisconsin organ transport solution on wet ice.

Each of the whole bladders collected from 4 dogs were used for the *in vitro* muscle strip contractility studies. Bladders were washed in Tyrode’s buffer (125 mM NaCl, 27 mM KCl, 4.2 mM NaH_2_PO_4_, 1.8 mM CaCl_2_, 5 mM MgCl_2_, 23.8 mM NaHCO_3_, and 5.6 mM dextrose), immersed in Custodiol^®^ HTK organ transport media, (5 mM NaCl, 9 mM KCl, 1 mM potassium hydrogen 2-ketoglutarate, 4 mM MgCl_2_, 18 mM histidine HCl, 180 mM histidine, 2 mM tryptophan, 30 mM mannitol, and 0.015 mM CaCl_2_) and saved on ice at 4°C for contraction studies performed the following day.

Dissections of all specimens were performed in a cold room (0–5°C), maintaining the tissues on ice during the dissections. Bladder muscle strips were dissected from the central middle part, at least 1 cm above the ureteral orifices. These dissections were performed using sharp micro scissors and 5x magnifying loops. The mucosa was separated from the underlying layers by sharp dissection, as well as from the peritoneal fat present in human bladders. Muscle strips were obtained with the long axis parallel to the direction of the visible muscle fiber bundles. Strips were clamped between force transducers and positioners and mounted in muscle baths (S1 Fig) containing 10 ml of Tyrode’s solution aerated with 95% O_2_ and 5% CO_2_ at 37°C.

Strips were initially stretched slowly to 20 mN of isometric tension and allowed to relax to approximately 10 mN of basal tension [30]. Although the relaxation response of the strips was not particularly tested, we did not observe any differences between strips during their pre-drug baseline responses. Previously it has been reported that the relaxation of human detrusor strips, evaluated using the β-adrenoceptor agonist, was not associated with gender, age, or passive tension (10 mN) and KCl-induced tone [31]. Contractile responses were monitored with isometric force transducers, as previously described [32]. Electrical field stimulation (EFS) of 8, 12 and 24 volts (V), 1 millisecond (ms) pulse duration and 30 Hertz (Hz) frequency was delivered to each strip using a Grass S88 stimulator (Natus Neurology, Inc., Warwich, RI) interfaced with a Stimu-Splitter II (Med-Lab Instruments, Loveland, CO) power amplifier and LabChart® software (ADInstruments). After a 30-minute equilibration, strips were exposed sequentially to an isotonic buffer containing 120 mM potassium chloride (KCl), which was immediately washed out after maximal responses were produced. Strips were then sorted out for each treatment group according to their responses to KCl such that the mean contractile response to KCl was the same between drug treatment groups. Expectedly, the average responses to KCl were not different between strips that were assigned for each treatment in either humans or dogs (S2 Fig). After re-equilibration for approximately 1 hour, trains of EFS of 1 ms pulse duration, 12 V, 8 Hz at 90 second intervals were applied to each strip for about 20 minutes.

Then, subsets of strips were incubated with either: 1) 100μM of hydrogen peroxide (H_2_O_2_, catalog # H325-500, Fisher Chemicals, East Bunker Court Vernon Hills, IL); 2) 100μM of apocynin, an inhibitor of Nox enzymes and ROS scavenger (catalog # 178385, Calbiochem-MilliporeSigma, Sigma-Aldrich Inc., St. Louis, MO); 3) 1μM of the Nox activator angiotensin II (Ang II, catalog # ALX-151039-M005, Enzo Life Sciences, Inc., Farmingdale, NY); or 4) 10μM of ZD7155 hydrochloride, an AT1 receptor specific antagonist (catalog # 1211, Tocris Bioscience, Minneapolis, MN), each for 20 minutes in continued trains of EFS. Next, the same subsets of strips treated first with antagonists (treatment #1) were treated with either H_2_O_2_ or Ang II (treatments #2 and/or #3). For that, subsets of strips were sequentially treated with: 1) apocynin (treatment #1) for about 20 minutes and then with either H_2_O_2_ (treatment #2) or Ang II (treatment #2) for similar time frames without washout of the apocynin; 2) apocynin (treatment #1) for about 20 minutes, then H_2_O_2_ (treatment #2), then followed by Ang II (treatment #3), each for about 20 minutes without washout of the earlier treatments; or 3) ZD7155 (treatment #1) for about 20 minutes, followed by Ang II (treatment #2) for about 20 minutes without washout of the ZD7155. Each treatment was for about 20 minutes, or when either the maximum contraction or maximum inhibition was achieved or when the tension returned almost to baseline levels in case of treatment with either H_2_O_2_ or Ang II. Responses to 30μM of the muscarinic receptor agonist bethanechol (catalog # 1071009, Sigma-Aldrich, Saint Louis, MO) were then determined in the continued presence of the previously added drugs (treatments #1─3), to test the viability of strips at the end of each experiment (S3 Fig). Responses to bethanechol were not different between strips that were subjected to different drug treatments in either humans or dogs. All responses were measured as tension and expressed in milli Newtons (mN).

As was previously reported, no sex differences were found in strips from male versus female humans [30]; therefore, data from these muscle strips were grouped together. Sex differences could not be examined in dog tissues because samples of convenience were used (i.e., only 1 female dog versus 3 males).

### Measurement of superoxide

Superoxide levels were measured in homogenized dog bladder muscle using lucigenin-enhanced chemiluminescence which shows the ability of the smooth muscle tissue to generate superoxide. For this, we used adjacent mucosa-denuded muscle segments of dog bladder tissue (obtained as described earlier). Briefly, mucosa-denuded muscle segments were cut into small pieces, transferred into cryovials, immediately flash-frozen using liquid nitrogen, and stored at -80°C until use. Each piece was ground into powder using a mortar and pestle. The sample was put into the mortar with a small amount of liquid nitrogen, after which once evaporated, the muscle piece was ground into powder. The powder was collected and transferred to a prelabelled eppendorf tube, and vortexed 3 times, for 1 min each, using 1X Hank’s Balanced Salt Solution (HBSS, catalog # 14175–095, Gibco, Thermo Fisher Scientific, Green Island, NY). This was performed without centrifugation since total muscle homogenates were required. The protein concentration of each sample was measured on each assay day for higher accuracy using a Pierce™ BCA Protein Assay Kit (catalog # 23227, Pierce, Rockford, IL). For the lucigenin assay, 10 mM of dark-adapted lucigenin (10,10’-dimethyl-9,9’-biacridinium, dinitrate, catalog # 14872, Cayman Chemicals, Ann Arbor, MI) was diluted in a sample buffer solution (0.8 mM MgCl_2_ and 1.8 mM CaCl_2_ in HBSS) to a final concentration of 25μM in the buffer solution. Primarily, lucigenin is a chemiluminogenic substrate that upon oxidation gives high yield of light-emitting products (photons) and chemiluminescence [33, 34]. In the wells of a white opaque 96 well microplate (Nunclon™ Delta Surface, Flat-Bottom Microplate, catalog # 136101, Thermo Scientific, DK-4000 Roskilde, Denmark), 25μl of each sample’s total homogenate were added into triplicate wells. Then, 115 μl of sample buffer without lucigenin and 40 μl of sample buffer with lucigenin were added to each well with a final lucigenin concentration after NADPH addition (see later text) at 5μM. The plate was then placed into a luminometer plate reader (GloMax® Discover Dual Injectors with Pumps, catalog # GM3030, Promega, Madison, WI) that had been warmed to 37°C. Basal levels were determined by measuring the light emitted from each well over a period of 15 min. Then, 20μl of 1mM NADPH (made fresh on each assay day; sodium salt, catalog # 9000743, Cayman Chemicals, Ann Arbor, MI) was injected into each well using the dual injectors to final concentration of 100μM (which triggers a high increase in ROS instantly after addition [35–37]. The plate was then read again over a 15-minute period. Next, 4μl of 1M Tiron solution (4,5-dihydroxy-1,3-benzene-disulfonic acid, a superoxide scavenger, catalog # ab146234, abcam, Waltham, MA) was added to each well using the dual injectors to a final concentration of 20 mM in each well, and the plate was re-read over a 15-minute period. The plate was run at 37°C during all plate reads. Reads are reported as relative light units (RLU) emitted over time (i.e., the photoemission was assayed). The amount of superoxide produced over time was calculated as follows: the values for light units obtained over the measured period for each run (basal, NADPH, and Tiron) were averaged so that for each well, there are 3 values per run. We divided these by 25 (since there was 25μl of sample per well) to get the mean light units (MLU) per μl, per well. We then divided by the protein concentration (microgram per μl) to calculate MLU per microgram of tissue. To then calculate the “actual increase in ROS”, we subtracted the basal value from the NADPH and Tiron values.

### Statistical analyses

Statistical analyses were performed using Prism version 9.4.1 (GraphPad Software, La Jolla, CA). Data are presented as mean ± 95% confidence intervals (CI). P-values were adjusted for multiple comparisons whenever applicable and values of 0.05 or less were considered statistically significant for all analyses. Numbers of human or animal specimens per group and per treatment (indicated as “N”), and the numbers of muscle strips per treatment (indicated as “n”), are listed in all figures. A repeated measures mixed-effects REML (Restricted Maximum Likelihood) model was used to compare treatment results, using the factors drug treatment (pre- versus post- treatment), and species (human versus dog). This was followed by Sidak’s multiple comparison post hoc tests to determine differences between groups. Adjusted p values are reported.

## Results

### Exogenous ROS, hydrogen peroxide (H_2_O_2_), enhanced EFS-induced bladder strip contractions in both species

**Fig 1:**
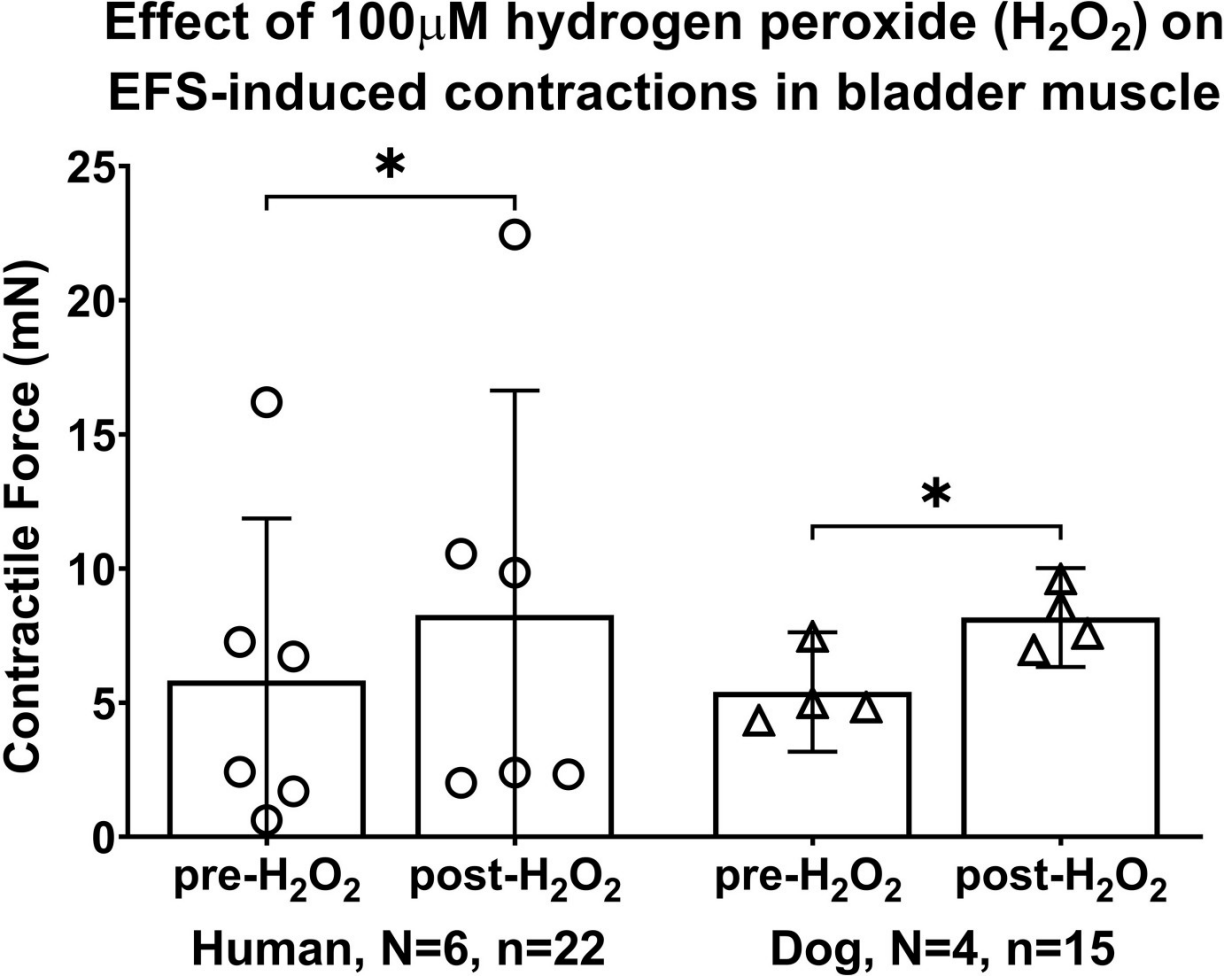
Exogenous hydrogen peroxide (H_2_O_2_, 100μM) enhances EFS-induced muscle strip contractions in human and dog bladders. The maximal responses are expressed in milli Newtons (mN). EFS = electrical field stimulation. Data is presented as mean ± 95% CI. *: p < 0.05, comparing pre- versus post-H_2_O_2_ treatment.

The effects of application of the ROS, hydrogen peroxide (H_2_O_2_), at the physiological concentration of 100μM, enhanced EFS-induced smooth muscle strip contractions similarly in both species in the mixed-effects statistical model (treatment effect, p = 0.004; species effect, p = 0.8). Post hoc analyses showed in human bladders that 100μM H_2_O_2_ enhanced EFS-induced smooth muscle strip contractions, compared to EFS-induced contraction before application of the H_2_O_2_ (5.8 ± 6.0 pre-H_2_O_2_ versus 8.3 ± 8.4 post-H_2_O_2_; p = 0.04, Fig 1). Similarly, in dog bladders, 100μM H_2_O_2_ increased the EFS-induced contractions (5.4 ± 2.2 pre-H_2_O_2_ versus 8.2 ± 1.8 post-treatment; p = 0.04, Fig 1). It has been known that different levels of H_2_O_2_ induce specific intracellular responses [38–40]. In our studies, H_2_O_2_ at a concentration of 100μM was added to the muscle bath as exogenous agent and maybe it isn’t produced physiologically in our in-vitro system. Although, we did not test smaller concentrations of H_2_O_2_, since the concentration that we tested occurs within the low range of H_2_O_2_ that has been indicated as a physiological concentration or as an optimal extracellular, sub-toxic, or non-lethal concentration [41–46]. In human airway epithelium, it has been shown that H_2_O_2_ at a concentration of 100μM enhances intracellular ROS production without affecting their viability, proliferation or morphology, while at the higher concentrations of 300 and 500μM, H_2_O_2_ significantly induces cell death hours after treatment [47]. For future studies, it may be of interest to test different concentrations of H_2_O_2_.

**Fig 2:**
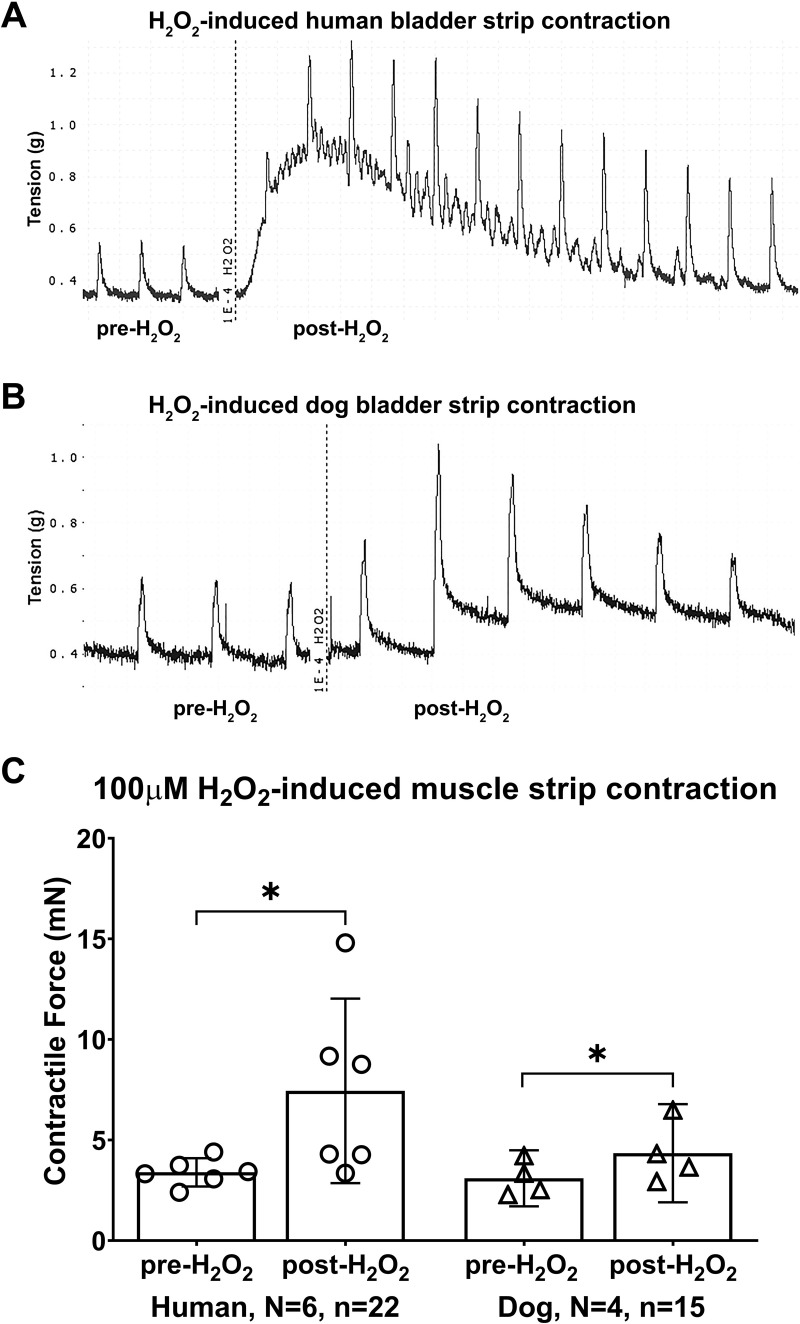
Bladder muscle strip responses to direct stimulation by 100μM H_2_O_2_. Representative tracings of H_2_O_2_-induced direct muscle strip contraction in human (A) and dog (B). Maximal responses to 100μM H_2_O_2_ in muscle strips from bladders of both species (C), comparing pre- versus post-H_2_O_2_ treatment. The maximal responses in (C) are expressed in milli Newtons (mN). Data is presented as mean ± 95%CI. *: p < 0.05, comparing pre- versus post-H_2_O_2_ treatment.

Also, the addition of H_2_O_2_ into the muscle bath caused direct strip contractions in bladder strips that were similar in both species (Fig 2A–2C; mixed-effects model: treatment effect, p = 0.02; species effect, p = 0.2). In the human bladder strips, the effect was about 2.3-fold higher than that acquired from the same strips under non-stimulated conditions (3.4 ± 0.7 pre-H_2_O_2_ versus 7.4 ± 5.7 post-H_2_O_2_; p = 0.04, Fig 2A and 2C). In the dog bladder strips, this effect was about 1.4-fold, compared to pre-H_2_O_2_ treatment (3.1 ± 1.4 pre-H_2_O_2_ versus 4.3 ± 2.4 post-H_2_O_2_; p = 0.04, Fig 2B and 2C).

### The NADPH oxidase (Nox) inhibitor and ROS scavenger, apocynin, attenuated EFS-induced bladder strip contractions in both species

**Fig 3:**
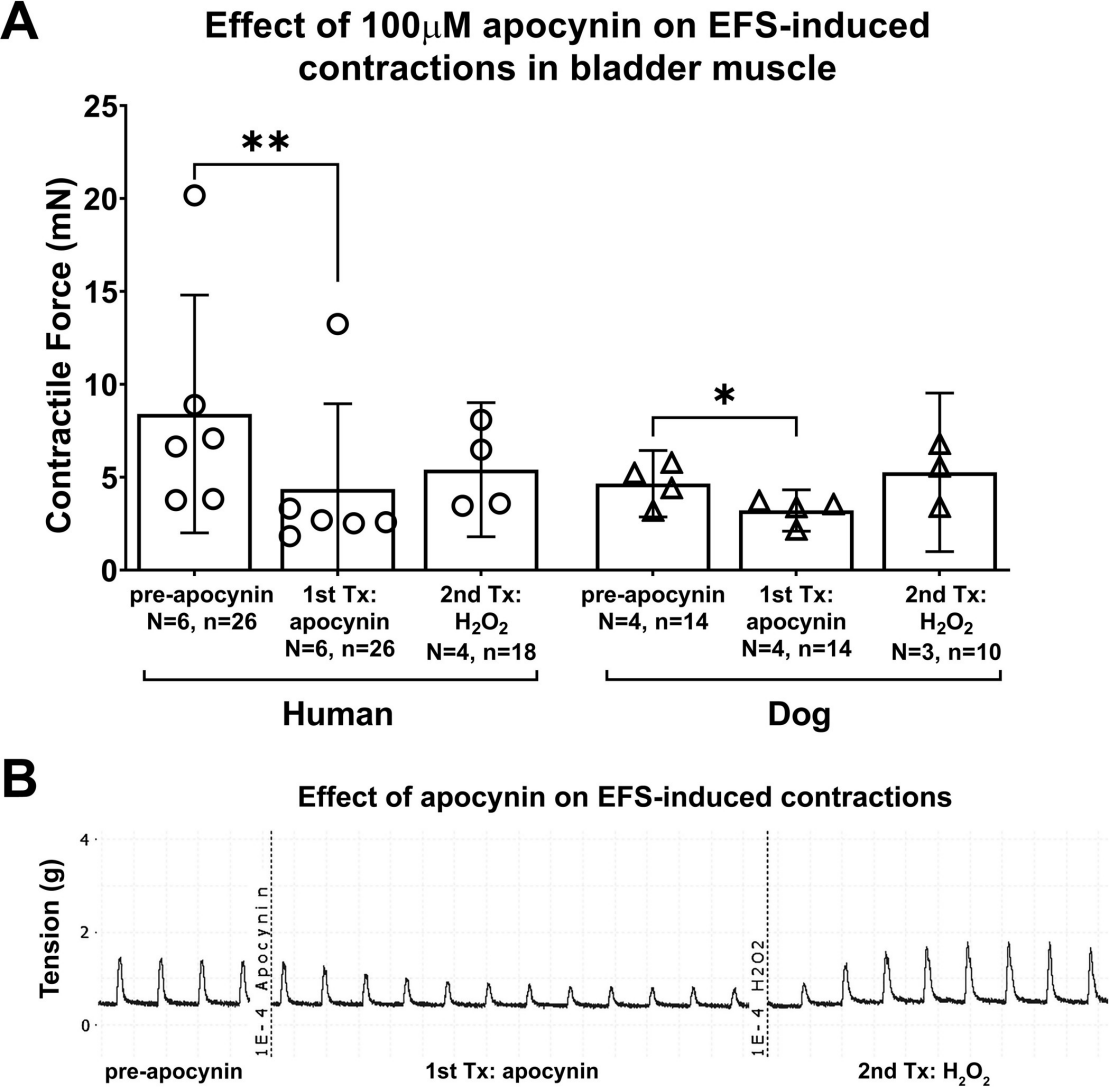
Nox inhibitor and ROS scavenger, apocynin, attenuated EFS-induced bladder muscle strip contractions in both species. The H_2_O_2_ (100μM, second treatment) was added to the muscle baths without washout of the apocynin (100μM, first treatment). (A) The maximal responses are expressed in milli Newtons (mN). (B) Representative tracing of the apocynin effect and then the H_2_O_2_ effect on EFS-induced human bladder muscle strip contraction. EFS = electrical field stimulation. Tx = treatment. Data is presented as mean ± 95%CI. *: p < 0.05 and **: p < 0.01, comparing post-apocynin versus either pre-apocynin, or H_2_O_2_ treatments.

We next examined the effects of inhibiting ROS generating enzymes using apocynin at the concentration of 100μM on EFS-induced muscle contractions. The mixed-effects statistical model showed a treatment effect (p = 0.04), yet no differences between humans and dogs (species effect, p = 0.5). Post hoc analyses showed that apocynin (100μM) attenuated intrinsic muscle strip activity in bladders from humans, compared to the strips’ pre-application results (8.1 ± 6.5 for pre-apocynin versus 4.2 ± 4.6 for post-apocynin; p = 0.008, Fig 3A). As described in the methods, this was followed by additional sequential treatment with H_2_O_2_ (a representative trace showing the treatment sequence is shown in Fig 3B). In the human bladder strips, treatment with H_2_O_2_ (treatment #2) following apocynin treatment (treatment #1) slightly enhanced EFS-induced muscle contraction (4.2 ± 4.6 for post-apocynin versus 5.4 ± 3.6 after treatment with H_2_O_2_; Fig 3A). This H_2_O_2_ treatment result did not differ significantly from either post- or pre-apocynin results (p = 0.6 and p = 0.3, respectively).

An attenuation of muscle strip activity was also observed after apocynin treatment in dog bladder muscle strips (4.7 ± 1.8 for pre-apocynin versus 3.2 ± 1.1 for post-apocynin; p = 0.01, Fig 3A). In the dog bladders, treatment with H_2_O_2_ (treatment #2) following apocynin treatment (treatment #1) slightly enhanced EFS-induced muscle contraction (3.2 ± 1.1 for post-apocynin versus 5.3 ± 4.3 after treatment with H_2_O_2_). The H_2_O_2_ treatment result did not differ significantly from either post- or pre-apocynin results (p = 0.7 and p = 0.3, respectively).

### Key Nox activator, angiotensin II (Ang II), increased EFS-induced contractions in dog bladder strips only

**Fig 4:**
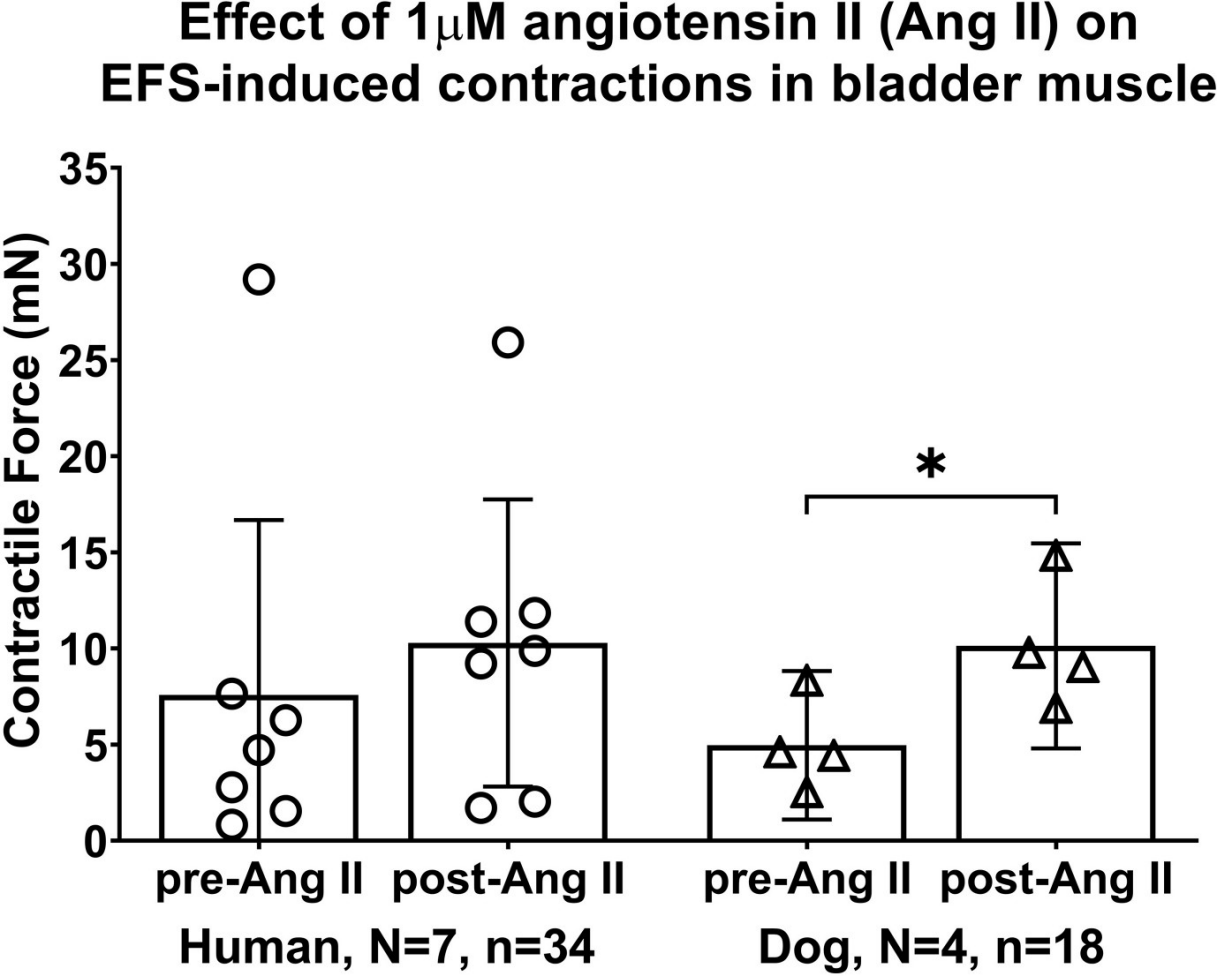
Angiotensin II (Ang II) enhances EFS-induced muscle strip contractions in dog bladders, but not humans. The maximal responses are expressed in milli Newtons (mN). EFS = electrical field stimulation. Data is presented as mean ± 95%CI. *: p < 0.05, comparing pre- versus post-Ang II treatment.

Additionally, we examined the effects of administration of a key Nox activator and a pro-inflammatory peptide Ang II (1μM), since it had been demonstrated that in vascular smooth muscle cells, Ang II increases H_2_O_2_ levels. The mixed-effects statistical model showed a treatment effect (p = 0.01), yet no differences between humans and dogs (species effect, p = 0.8). However, in human bladders, the post hoc analyses showed that application of Ang II (1μM) did not significantly enhance EFS-induced contractions (7.6 ± 9.1 for pre-Ang II versus 10.3 ± 7.5 for post-Ang II, p = 0.1, Fig 4). In contrast, in dog bladders, Ang II enhanced the EFS-induced contractions (5.0 ± 3.9 for pre-Ang II versus 10.1 ± 5.3 for post-Ang II, p = 0.03, Fig 4).

We sought to find a difference in the human versus dog results. Knowing that there were age differences between the human donors (which ranged between 22 and 57 years) and dog donors (which were of similar age), we correlated the Ang II EFS-induced contraction results with donor age. We found that the post Ang II treatment results from the human bladder muscle strips correlated strongly and negatively with the age of the donor (r = -0.82, p = 0.01). There was no correlation in the dogs.

### Ang II increased direct muscle strip contractions significantly in human bladder strips

**Fig 5:**
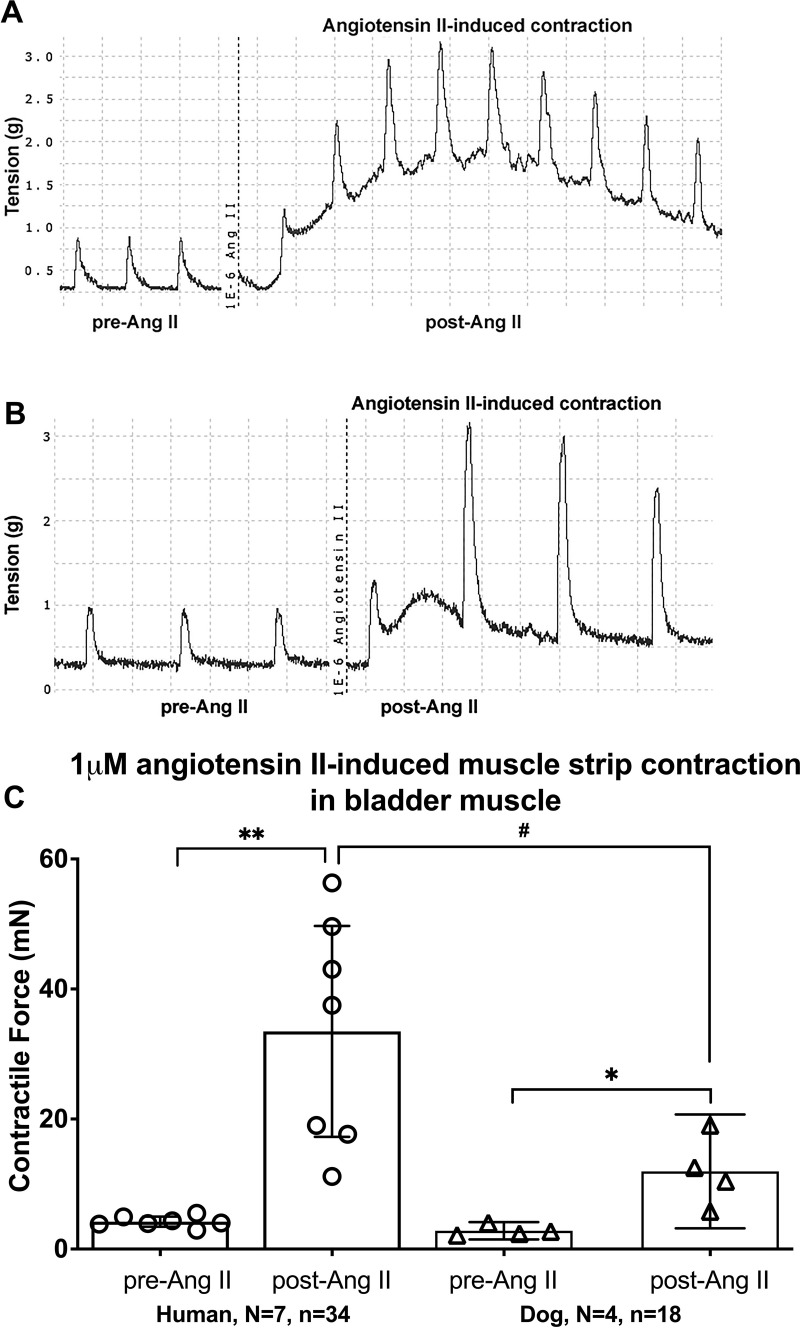
Bladder muscle strip responses to a direct stimulation by 1μM angiotensin II (Ang II). Representative tracing of Ang II-induced direct muscle strip contraction in human (A) and dog (B). Maximal responses to 1μM Ang II in muscle strips from human and dog bladders, comparing pre- versus post-Ang II treatment. The maximal responses in (C) are expressed in milli Newtons (mN). Data is presented as mean ± 95%CI. *: p < 0.05 and **: p < 0.01, comparing pre- versus post-Ang II treatment; ^#^: p < 0.01, comparing post-Ang II treatment between humans and dogs.

In contrast to the above results, Ang II greatly induced direct muscle strip contractions in bladder strips. The mixed-effects statistical model showed a treatment effect (p = 0.0006), a species effect (p = 0.02), as well as a treatment x species effect (p = 0.04). The post hoc analyses showed in the human bladder muscle strips that Ang II greatly augmented direct muscle strips’ contractions (4.2 ± 0.8 for pre-Ang II versus 33.5 ± 16.2 for post-Ang II, p < 0.0001, Fig 5A and 5C). Ang II also caused contractions of dog bladder muscle strips (2.8 ± 1.3 for pre-Ang II versus 12.0 ± 8.7 for post-Ang II, p = 0.04, Fig 5B and 5C). The post-treatment effects of Ang II were statistically significantly different between human and dog bladder muscle strips (p = 0.004).

### Ang II treatment after apocynin treatment enhanced EFS-induced contractions in both species

**Fig 6:**
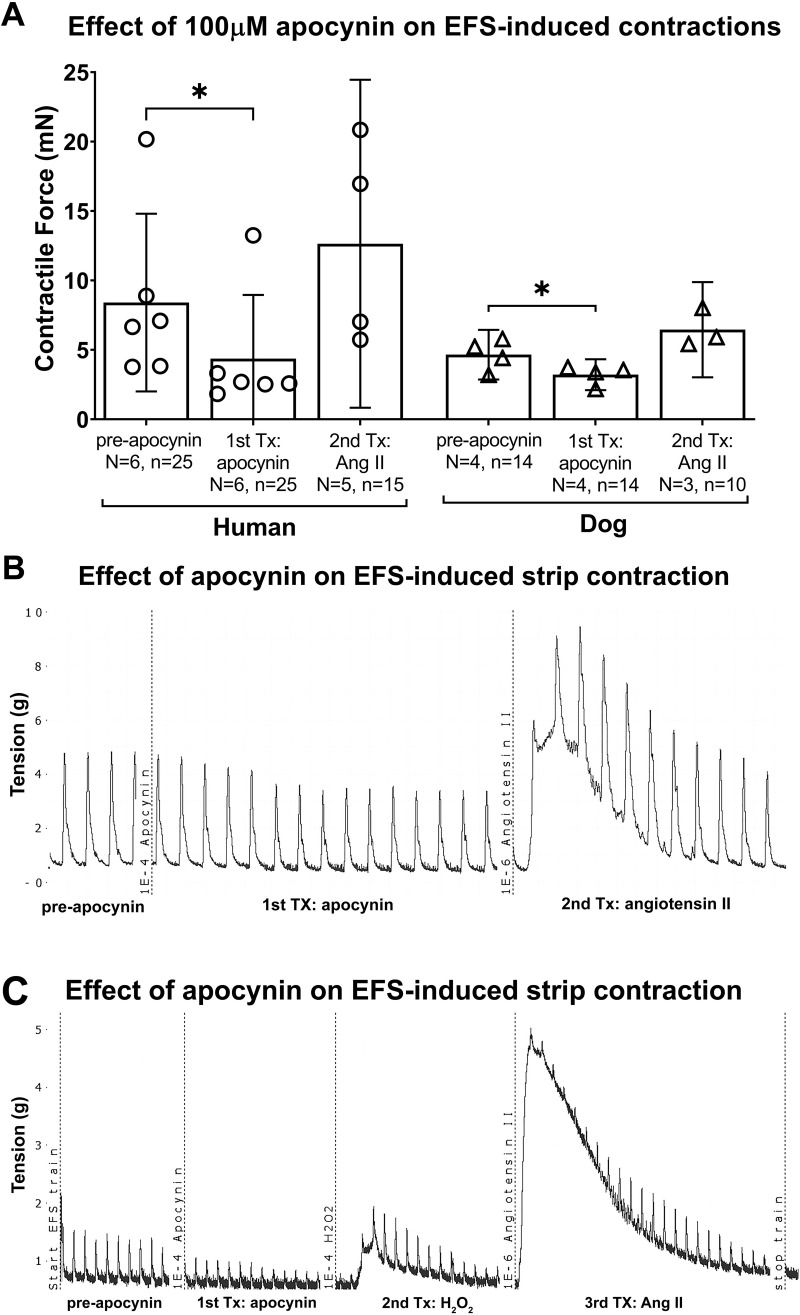
Angiotensin II treatment, after apocynin, recovered EFS-induced muscle strip contractions in human and dog bladders. (A) The maximal responses are expressed in milli Newtons (mN). (B) Representative tracing of apocynin effect and then Ang II effect on EFS-induced human bladder muscle strip contraction. (C) Representative tracing of apocynin (first treatment) effect, then H_2_O_2_ (second treatment), and then Ang II (third treatment) effect on EFS-induced human bladder muscle strip contraction. In each, the subsequent muscle bath treatments occurred without washout of prior treatment(s). EFS = electrical field stimulation. Tx = treatment. Data is presented as mean ± 95% CI. *: p < 0.05, comparing responses to post-apocynin versus either pre-apocynin, or Ang II treatments.

We further explored the effects of treatment with Ang II added sequentially after apocynin treatment, or apocynin and then H_2_O_2_ treatment, before the Ang II treatment (Fig 6A–6C, with representative tracing of the treatment sequence shown in Fig 6B and 6C). The mixed-effects statistical model showed a treatment effect (p = 0.04), yet no species effect (p = 0.2) or treatment x species effect (p = 0.2). The post hoc analyses showed a pre- versus post-apocynin treatment effect of depression of the EFS-induced strip contractions (Fig 6), similar to that seen in Fig 3‘s experiment. Yet, the secondary treatment with Ang II showed an enhanced EFS-induced strip contractions that was back to the control levels, compared to post-apocynin treatment in both human (12.6 ± 11.8 versus 4.4 ± 4.6, p = 0.4) and dog (6.5 ± 3.4 versus 3.2 ± 1.1, p = 0.3) bladders (Fig 6A). A similar recovery from the apocynin reduced contractions was observed after the Ang II treatment, regardless of whether H_2_O_2_ was included prior to the final Ang II treatment or not (see representative traces in Fig 6B and 6C).

### The AT1 receptor-specific inhibitor, ZD7155, attenuated EFS-induced contractions

**Fig 7:**
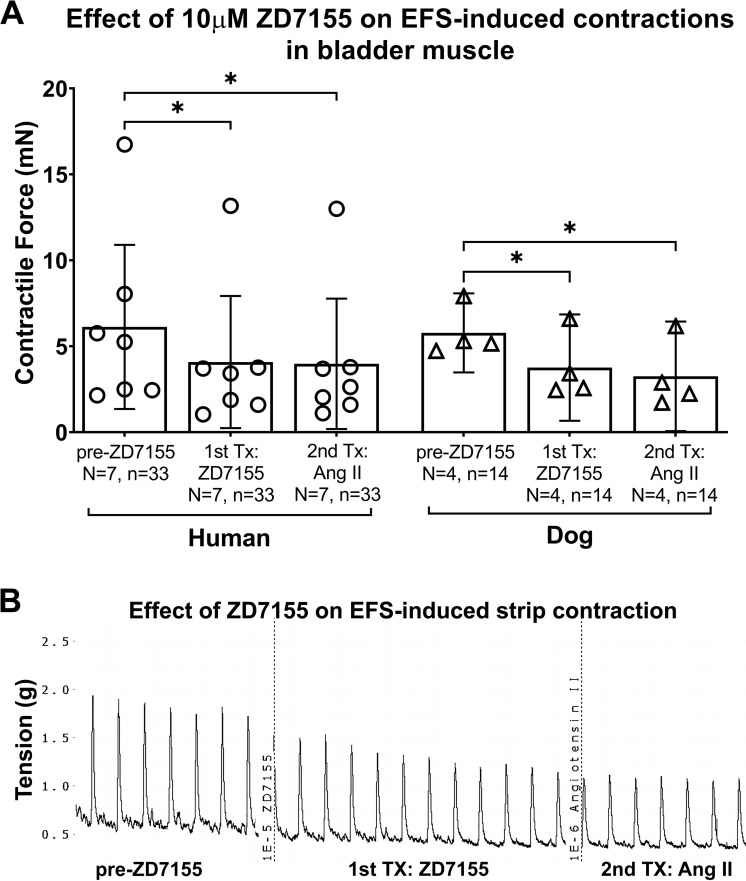
AT1 receptor specific inhibitor, ZD7155, attenuated EFS-induced muscle strip contractions in human and dog bladders. Angiotensin II (Ang II, 1μM, second treatment) was added to the muscle baths without washout of the ZD7155 (10μM, first treatment). (A) The maximal responses are expressed in milli Newtons (mN). (B) Representative tracing of ZD7155 effect and then Ang II effect on EFS-induced human bladder muscle strip contraction. EFS = electrical field stimulation. Tx = treatment. Data is presented as mean ± 95%CI. *: p < 0.05, comparing post-ZD7155 versus either pre-ZD7155, or Ang II treatments.

We next examined the effects of administration of AT1 receptor specific antagonist, ZD7155 (10μM) on EFS-induced activity. The mixed-effects statistical model showed a treatment effect (p = 0.0007), but no species difference (p = 0.8) or species x treatment effect (p = 0.8). The post hoc analyses showed that in humans, as well as in dogs, that treatment with ZD7155 (10μM) inhibited EFS-induced activity, compared to pre-treatment in bladders, as shown in Fig 7A (human bladders: 6.1 ± 4.8 for pre-ZD7155 versus 4.1 ± 3.8 for post-ZD7155, p = 0.03; dog bladders: 5.8 ± 2.3 for pre-ZD7155 versus 3.7 ± 3.1 for post-ZD7155, p = 0.01). In both species, subsequent treatment with Ang II (treatment #2) did not restore muscle contractions following the ZD7155 treatment (treatment #1) (p = 0.8 and p = 0.1, respectively) (Fig 7A and 7B).

### ROS levels in dog bladder muscle tissue

**Fig 8:**
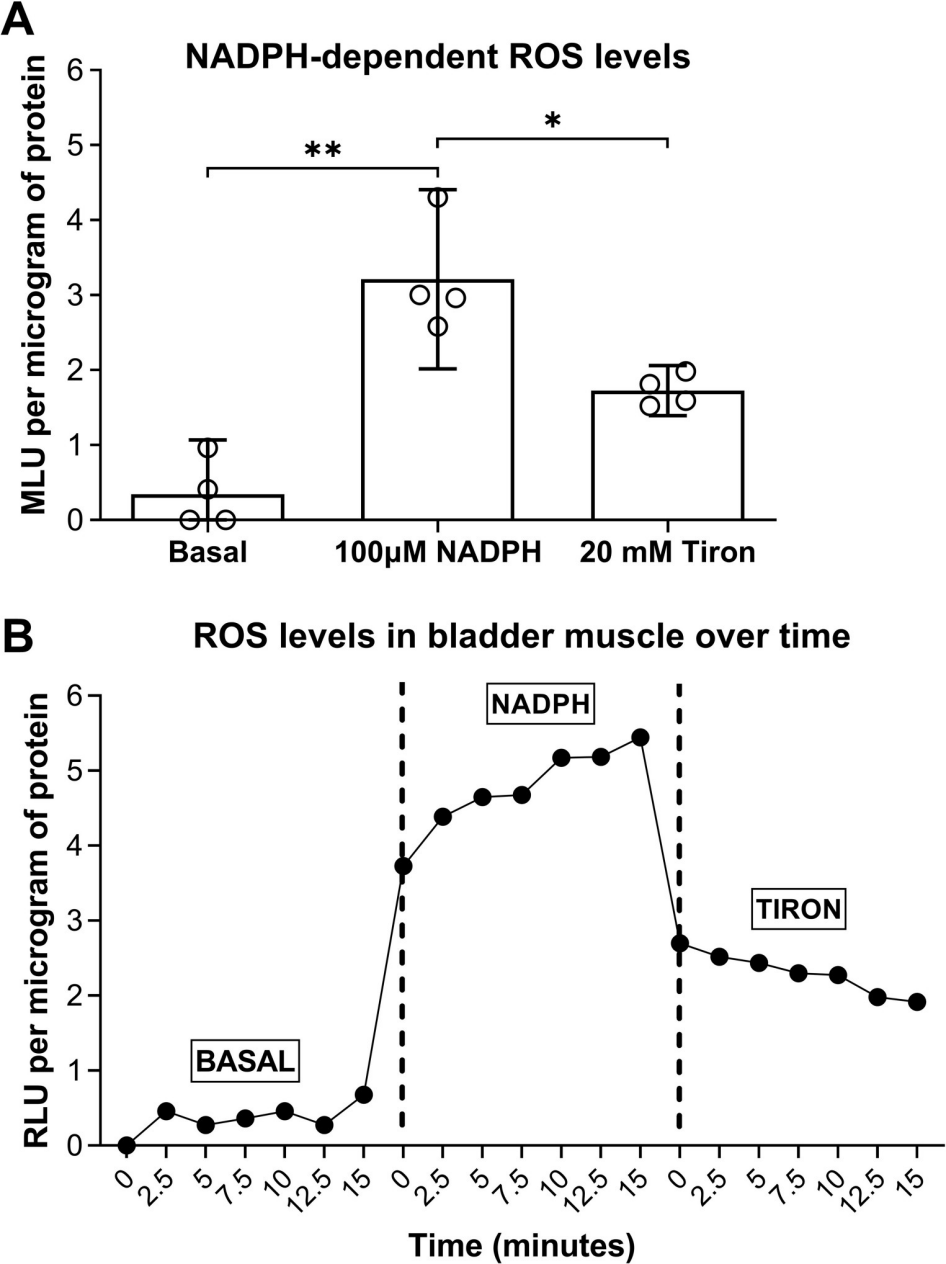
NADPH enhanced ROS, and specifically, superoxide levels in dog bladder smooth muscle using lucigenin-enhanced chemiluminescence. (A) Total muscle homogenates were exposed to dark-adapted lucigenin in balanced salt solution and baseline was measured (Basal). Superoxide production was enhanced in the presence of NADPH (100μM). Superoxide production was attenuated by the addition of 20 mM Tiron. (B) Representative photon emission in response to the 3 different conditions (Basal, NADPH, and Tiron) measured in a luminometer over time (in minutes). MLU = mean light units. RLU = relative light units. Data is presented as mean ± 95%CI. *: p < 0.05, NADPH versus baseline or Tiron.

Adjacent dog bladder muscle samples were prepared as total homogenates for lucigenin-enhanced chemiluminescence assays. We found that the addition of NADPH (100μM) to lucigenin-containing buffer enhanced the lucigenin signal in the samples over background by stimulating ROS production (p = 0.001, Fig 8A and 8B). The ROS levels in response to the superoxide scavenger, Tiron (20 mM), were lower than that elicited by NADPH (p = 0.04, Fig 8A), suggesting that NOX is a significant source of superoxide in dog bladder muscle in response to NADPH exposure.

## Discussion

### Summary of objective and results

Utilizing *in vitro* studies, we aimed to explore the physiological role of ROS/ Nox in regulating muscle function in bladders collected from humans and dogs with no known bladder pathologies. The exogenous ROS, H_2_O_2_, enhanced EFS-evoked contractions (and also directly enhanced muscle strip contractions), while the Nox inhibitor and ROS scavenger, apocynin, attenuated the EFS-induced contractions. Treatment with H_2_O_2_ following apocynin treatment improved the EFS-induced contractions. The enhancement of EFS-evoked contractions by H_2_O_2_ and the inhibition of these contractions by apocynin demonstrates the functional relevance of ROS in regulating human and dog bladder smooth muscle activity and suggests that endogenous Nox-derived ROS regulates smooth muscle function. The Nox activator and inflammatory mediator, Ang II, known to act via the AT1 receptor [48, 49], enhanced the EFS-induced contractions in dog, but not human bladder muscle strips (Fig 4), yet induced direct strip contractions in both species (Fig 5). Also, treatment of apocynin-treated strips with Ang II restored EFS-induced contractions in both species. Blockade of the AT1 receptor using a specific inhibitor, ZD7155, reduced the EFS-induced contractions in both species. The augmentation of contractions by Ang II suggests that activation of Nox via a receptor’s ligand can also enhance smooth muscle activity, while the inhibitory effect of the selective antagonist ZD7155 indicates that the effect of Ang II is mediated by the AT1 receptor.

### Effects of the exogenous ROS, H_2_O_2_ on EFS-induced contractions

We observed enhanced EFS-induced contractions by the exogenous ROS, H_2_O_2_ in strips from both human and dog bladders (Fig 1). Similarly, H_2_O_2_ treatment enhances EFS-induced contraction of cat tracheal strips [18], and isolated rat bronchi [22], and application of a superoxide generating compound, pyrogallol, enhances EF-induced contraction of rat mesenteric arteries [50]. H_2_O_2_ treatment potentially leads to increased sensitivity to trains of EFS through the stimulation of intramural nerves and membrane-bound receptors. For example, EFS can excite detrusor smooth muscle strips directly by the release of neurotransmitters acetylcholine and/or ATP, dependent on the species [51, 52]. Nerve-evoked contractions at frequencies of 8–32 Hz appear to occur predominately by the response to released acetylcholine in urinary bladders [53]. The amount of the acetylcholine released endogenously from postganglionic nerves during EFS is frequency-dependent and correlates with the observed contractile force of detrusor muscle [54].

Even though H_2_O_2_ is a naturally occurring oxidative compound [55], it can cause damage to cellular and intracellular components [1, 20]. High concentrations of H_2_O_2_ (exceeding 300μM) may damage smooth muscle contractile protein function and cause a net reduction of contraction despite raising intracellular calcium concentrations [56]. In muscle strips from rabbit bladders, Francis and colleagues showed that contractile responses of rabbit bladder strips to field stimulation, at the frequencies of 2, 8, and 32 Hz, were decreased by increasing H_2_O_2_ concentrations [57]. An inhibitory effect on contractility was observed at H_2_O_2_ concentrations exceeding 10 mM, presumedly due to a loss of sensitivity of the contractile proteins to calcium that would reduce net muscle contractility. Additionally, a study in pigs revealed that bladder smooth muscle tissues become susceptible to oxidative stress induced by ROS (Cumene hydroperoxide, 0.1–0.8 mM, lipophilic hydroperoxide). This effect was proposed to involve muscarinic receptor destruction and consequently, a reduction in strip contraction, yet no apparent effect on the cholinergic nerves as they still responded to the electrical stimulation at the frequencies used (8 and 32 Hz) [53]. Our lower H_2_O_2_ concentration of 100μM appears to have avoided potential damaging effects of H_2_O_2_ since we saw enhanced muscle strip responses to EFS after H_2_O_2_ treatment. H_2_O_2_ enhanced responses to EFS have also been previously reported in tracheal strips of cat [18]. In our study and the Bauer et al. study, perhaps the concentration of H_2_O_2_ used had no damaging effect on the cellular and intracellular components, or the potentially damaged structures remained functional even if they exhibited mild susceptibility to the externally added H_2_O_2_.

The H_2_O_2_ may also have stimulated an increase in calcium levels, which in turn enhances maximal responses to EFS. A relationship between ROS and intracellular calcium levels has been documented [58], with increasing concentrations of H_2_O_2_ leading to increases levels of intracellular calcium in human and rat endothelial cells [59]. H_2_O_2_ can induce changes in intracellular calcium through oxidative modification of calcium channels or other proteins involved in calcium signaling [16, 60]. It was reported in cat trachea that the increased intracellular calcium produced by H_2_O_2_ was associated with a slow increase in baseline muscle tension and augmentation of EFS-evoked contractions [18]. We cannot rule out such effects of H_2_O_2_ during EFS that involve nerve activity and receptor activation, since these were not examined in our experiments.

Other possible reasons for our observed enhancement of EFS-induced and direct contractions of the muscle strips by H_2_O_2_, rather than the inhibition seen in the Francis et al. study in rabbit bladder [57] could be the different stimulation parameters used. The setup of the EFS train in the Francis et al. study was different from what we used in our study (50 V versus 12 V stimulus intensity, 2 ms versus 1 ms pulse duration, 10 versus 90 sec for the inter-train interval, frequencies of 2, 4, 8, 16, and 32 Hz versus only one frequency of 8 Hz). The 12 V used in our study was enough to produce maximal tension, yet apparently low enough to avoid any tissue fatigue during the 20 min of testing. Also, we used mucosa-denuded human and canine detrusor strips rather than full thickness rabbit bladder strips used in the Francis et al. study. We also only examined the effects of a single dose of H_2_O_2_ (100 μM) on EFS-induced contractions.

### Direct effects of H_2_O_2_ on muscle strip contractility

The direct effect of H_2_O_2_ (Fig 2A–2C) is likely due to intracellular oxidative effects [55] and suggests that this reactive oxygen radical is playing a role in the contractility of human and dog bladder smooth muscle strips, as has been shown in rats [61]. Also, H_2_O_2_ has been shown to contract dog lung strips, bovine trachealis muscle strips [21], and rat airways [22]. These results indicate that H_2_O_2_ did not affect the ability of strips to produce maximal force, as previously reported [56]. A study in pigs revealed that bladder smooth muscle tissues can become susceptible to oxidative stress induced by ROS, and that the effect was attributed to muscarinic receptor destruction and consequently reduction in strip contraction [53]. This stress may explain the gradual decline in contractility of the muscle strips with continued exposure to H_2_O_2_ in both human and dog bladders.

### Effects of apocynin on EFS-induced muscle contractility

In both human and dog bladders, EFS-induced muscle contractility was attenuated by treatment of muscle strips with apocynin (Fig 3). Apocynin is an inhibitor of Noxs and a scavenger of non-radical oxidant species, such as H_2_O_2_ [62–65]. Apocynin inhibits the assembly of Nox responsible for ROS production [66], an inhibition that reduces intracellular ROS generation by Nox [67]. Furthermore, it has been reported that the Nox enzyme responsible for ROS production is fundamentally inactive in resting conditions, and that EFS elicits increases in ROS that can be blocked by apocynin [68, 69]. Our data and these past findings combined suggest that under physiological conditions, some ROS are released endogenously, in part from Nox enzyme activity, that then contribute to the normal EFS amplitude so that when apocynin is added, this endogenously and spontaneously released H_2_O_2_ or other superoxide is suppressed (different studies reported the additional off-target effects of apocynin as a scavenger of non-radical oxidant species) [62, 63]. Apparently, apocynin can modulate strip function and ROS activity via multiple mechanisms speculated as its actions [65]. With its lack of specificity, it is reasonable to anticipate that the inhibition of EFS by apocynin does not specify it as the only evidence for the role of Nox in regulating bladder muscle function. Thus, endogenous ROS generated from Nox enzymes can regulate smooth muscle function and modulate key bladder functions without exogenous stimuli, further evidence for the functional significance of ROS in bladder function.

We next examined if H_2_O_2_ treatment following apocynin treatment could counteract the apocynin attenuation of EFS-induced contractions (Fig 3). We found that treatment with H_2_O_2_ following apocynin treatment restored the apocynin suppressed EFS-induced muscle strip contraction, consistent with reduced ROS production due to the inhibitory effect of apocynin on Nox enzymes which can be restored by supplementing exogenous ROS, which could suggest that H_2_O_2_-induced contraction did not involve Nox activation. The exogeneous ROS (H_2_O_2_) bypasses the Nox activation and directly acts on the contractile machinery. On the other hand, the inability of apocynin to counteract the effect of H_2_O_2_ shows that apocynin does not act as a non-specific antioxidant against H_2_O_2,_ rather it acts as an inhibitor to directly suppress Nox activity. However, Figs 1 and 2 show that H_2_O_2_ enhances muscle contraction (both EFS and direct), as has another study using rat bladder [61]. Next, due to the specificity of apocynin as an inhibitor of Nox [64], and based on findings by this study and others that EFS elicits increases in ROS that are blocked by apocynin [68, 69], we suggest that the initial effects of apocynin treatment shown in Fig 3A is the suppression of endogenous ROS from the Nox enzymes, and that the response to an exogenous application of H_2_O_2_ then bypasses this inhibition and mirrors that seen without apocynin pre exposure (Fig 1). We suggest that these results confirm the involvement of extracellular ROS in activating the contractile machinery in strips of human and dog bladders, and that the muscle strips are subjected to ROS and Nox regulation.

### Effects of Ang II on EFS-induced muscle contractility

The observed enhancement of contractile responses to EFS by Ang II (1μM) in dog bladder strips (Fig 4) is in agreement with findings of previous *in vitro* experiments on urinary bladder smooth muscle from several species that revealed that Ang II is a potent contractile agent in this tissue [70–74]. In addition, the enhancement of contractile responses to EFS by Ang II has been well documented in vascular tissues of several species [50, 75–79]. Ang II is known as a potent stimulator of vascular ROS generation and that the potentiation of EFS-induced contractions by Ang II is mediated by superoxide production [80–83]. Additionally, it had been demonstrated that in vascular smooth muscle cells, Ang II increases H_2_O_2_ levels indirectly through the activation of Nox enzymes and consequently superoxide anion production [23, 64, 84–87], and that the effect of superoxide is mediated, at least in part, through its conversion to H_2_O_2_ [23, 37, 88, 89]. Surprisingly and different from dogs, Ang II did not enhance EFS-induced contractions in human bladders (Fig 4). It is worth noting that besides the natural variations between human subjects (we examined 7), we had a much wider variation in ages in the human donors (22 to 57 years) than in the dogs which were of similar age (6–8 months). The post Ang II treatment results correlated strongly and negatively with the age of the human donor (r = -0.82). Thus, many variables, including age, might contributed to the difference in the effect of Ang II on EFS in these two groups.

### Direct effects of Ang II on muscle strip contractility

Bath-applied Ang II (1μM) caused an increase in strips’ basal tension that reached a peak before gradually declining in continued exposure to Ang II in both human and dog bladders (Fig 5A and 5B). In isolated human detrusor muscle, it has been reported that in the continued presence of Ang II, desensitization of the functional response occurs, and that repeated administration of Ang II after a previous administration fails to initiate contractions (i.e., tachyphylaxis) [70, 90]. Therefore, to avoid such condition and to minimize errors during data interpretation, we only used a single dose of Ang II (1μM) per single muscle strip. The mean contractions of humans and dog strips following Ang II application (Fig 5C) matches results of several *in vitro* studies showing that Ang II causes contraction of human, canine, and rabbit bladder muscle [70, 72, 91, 92]. It is well documented that exposure to Ang II mediates its muscle contractile effect, at least in part by stimulating the activation of smooth muscle Nox enzymes implicated in mediating Ang II effect by the generation of ROS, and consequently superoxide production [23, 37, 82, 93]. Changes in intracellular calcium concentration have also been proposed as the main mechanism involved in the regulation of Ang II-induced smooth muscle contraction [94, 95].

We show here for the first time that the suppressive effects of apocynin on EFS-induced muscle contractions can be restored by Ang II treatment (Fig 6). We also observed the enhancement of direct contractions after the addition of Ang II following apocynin treatment. Studies have shown the excitatory effect of Ang II on EFS-induced muscle contractions is eliminated by apocynin [50, 96]. Also, contractility studies of human blood vessels showed that enzymes other than Nox play a role in Ang II-induced superoxide production, since inhibitors of these enzymes blunted Ang II mediated EFS-induced contractions [93, 97]. In addition to the possible role of superoxide, several other possible mechanisms may mediate the effects of Ang II on EFS-induced contractions, including neurotransmitter biosynthesis [98] and release [99], and/or neurotransmitter reuptake blockade [100]. Collectively, these results suggest that Ang II can act as both activator and enhancer of bladder smooth muscle contractile activity. This effect is partly mediated via Nox-derived ROS production, another novel finding of this study.

### Effects of AT1 receptor specific antagonist, ZD7155, on EFS-induced muscle contractility

The observed inhibition of EFS-induced contractions in both human and dog bladders by the AT1 receptor specific antagonist, ZD7155 (10μM; Fig 7) is in line with what has been reported in adult mammalian cells—that the physiological effects of Ang II are achieved mainly by binding directly to, and activating the receptor subtype AT1 [49, 50, 101]. These responses are supported by findings showing the presence of AT1 receptor in the detrusor smooth muscle layers of different species [48, 70, 90, 91, 102, 103]. The inhibited EFS-induced activity upon AT1 receptor antagonism suggests the possibility of local Ang II formation within detrusor muscle cells, as previously reported [104]. Since Ang II is a potent ligand for AT1 receptor, one might expect it to reverse the ZD715 blockade, however, the concentration that we used for Ang II was only 1μM, while that for ZD7155 was 10μM, given the affinities of both ligands to the receptor and assuming competitive antagonism, the antagonistic effect is prevailing. The non-responsiveness to Ang II application after ZD7155 treatment in strips isolated from human and dog bladders (Fig 7) further confirms that Ang II-induced contractions in human and dog detrusor strips is mediated through the AT1 receptor and supports specific action on AT1 receptor.

### Enhanced ROS levels in dog bladder muscle

Superoxide was measured in dog bladder muscle tissue using lucigenin-enhanced chemiluminescence, as this method was shown to be a sensitive for superoxide detection [34, 105, 106]. The observed enhancement in ROS production (Fig 8) is in agreement with previously reported work [107]. Thus, this data suggests that the enhanced chemiluminescence in response to NADPH is mediated by superoxide because the scavenger of superoxide Tiron was able to inhibit this response [107].

There are limitations to our study. We did not measure ROS production in human or dog bladder strips, in the absence or presence of apocynin and/or Ang II to support the functional data obtained in muscle baths because that was beyond our scope of study and not feasible using our muscle bath methodology (shown in S1 Fig). Also, commercially available and most widely used methods to measure peroxide or assess ROS are shown to be open to artefacts, as recently reported [108]. Thus, results obtained from using such methods should be interpreted with caution. Additionally, while ROS detectors, such as the free radical analyzer or microelectrode sensor, might be good tools to measure ROS, it is not feasible to use those methods in our system due to the larger volume of buffer that we add into a muscle bath (10 ml) relative to the low levels of ROS that are produced (picomolar to low micromolar). Please see S1 Fig. that shows the muscle strip mounted in a muscle bath. Also, most ROS are short-lived (lifespans of milliseconds or less), so it is hard to be certain of the amounts measured [108].

The other limitation is that we did not measure the superoxide levels in human bladder muscle tissue. However, we did not want to rely our conclusions exclusively on lucigenin data because the muscle strip contractility results related more to bladder muscle function and support the conclusions that the induced muscle contraction are mediated by ROS production, specifically by enhanced superoxide levels.

## Conclusion

Collectively, these data provide evidence for the functional significance of Nox-derived ROS in human bladder and that ROS can modulate bladder function without exogenous stimuli. The excitatory effects of angiotensin II on bladder smooth muscle function may have significant pathological implications since inflammation is an important mechanism associated with oxidative damage.

## Supporting information

S1 Fig Isolated bladder muscle tissue strip mounted in a muscle bath. Representative photograph shows a strip clamped between force transducers and positioners and mounted in a muscle bath containing 10 ml of Tyrode’s solution aerated with 95% O_2_ and 5% CO_2_ at 37°C. The size of the bath, relative to the ruler, is also shown.
(TIF)

S2 Fig Maximal contractile responses to potassium chloride in muscle strips of human and dog bladders. Responses to 120 mM potassium chloride (KCl) in different strips assigned to each treatment. All drug treatments applied later are indicated on the X-axis in either human or dog strips. The maximal responses to 120 mM KCl are expressed in milli Newtons (mN). N = number of bladders per group. n = number of strips per treatment. Data is presented as mean ± 95% CI.
(TIF)

S3 Fig Maximal muscle strip contractile responses to muscarinic receptor agonist, bethanechol, in human and dog bladders. Responses to 30μM bethanechol in different strips that were already subjected to the indicated drug treatment(s). All drug treatments that were added before bethanechol treatment are indicated on the X-axis in either human or dog strips. The maximal responses to 30μM bethanechol are expressed in milli Newtons (mN). N = number of bladders per group. n = number of strips per treatment. Data is presented as mean ± 95% CI.
(TIF)
