# Supplementary figures and images for: Mechanisms involved in nicotinamide adenine dinucleotide phosphate (NADPH) oxidase (Nox)-derived reactive oxygen species (ROS) modulation of muscle function in human and dog bladders
Source: PLoS One. 2023 Jun 23;18(6):e0287212. doi: 10.1371/journal.pone.0287212 (PMC10289437; doi:10.1371/journal.pone.0287212)

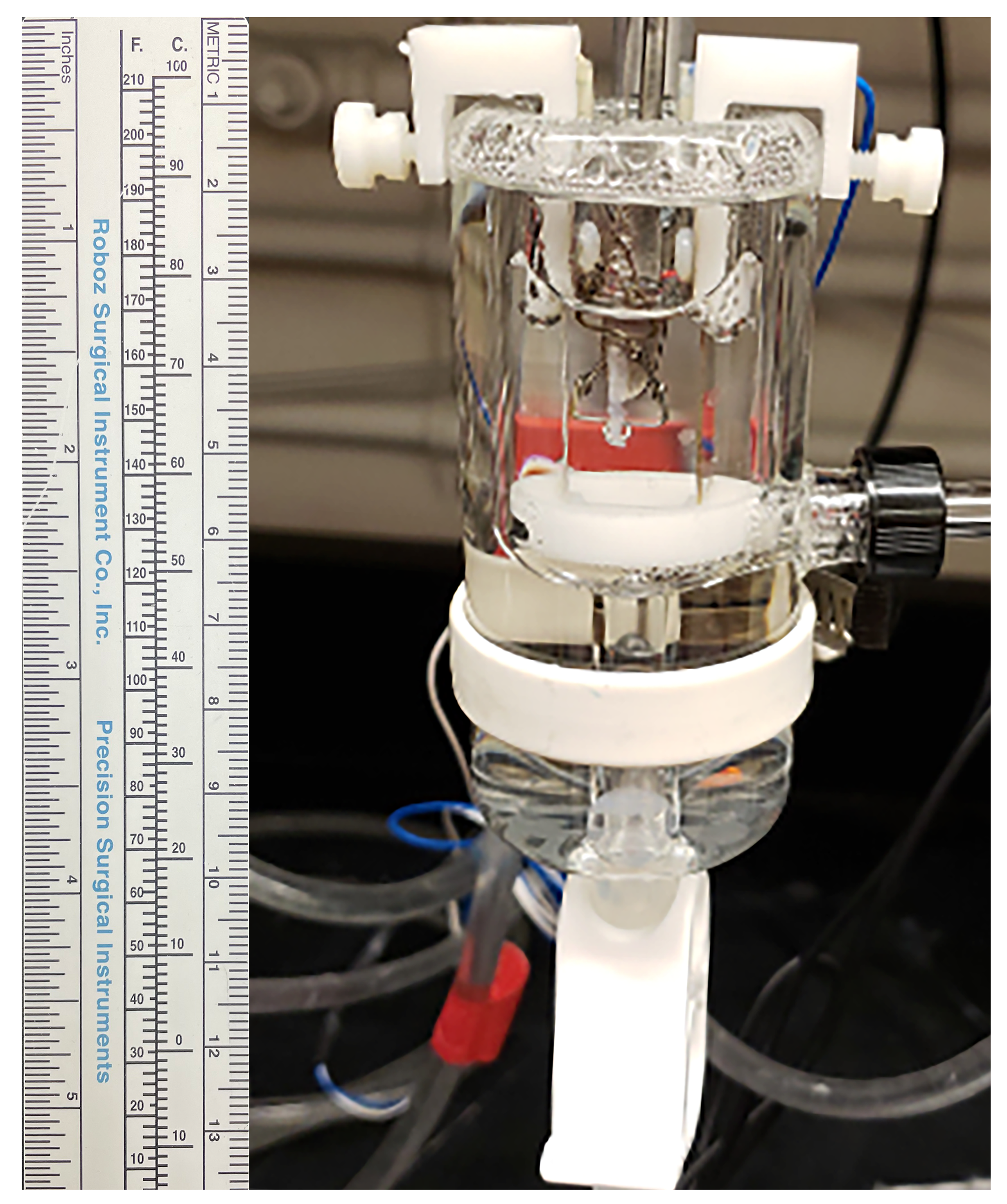

Supplement: S1 Fig — Representative photograph shows a strip clamped between force transducers and positioners and mounted in a muscle bath containing 10 ml of Tyrode’s solution aerated with 95% O2 and 5% CO2 at 37°C. The size of the bath, relative to the ruler, is also shown. (TIF) [file pone.0287212.s001.tif]

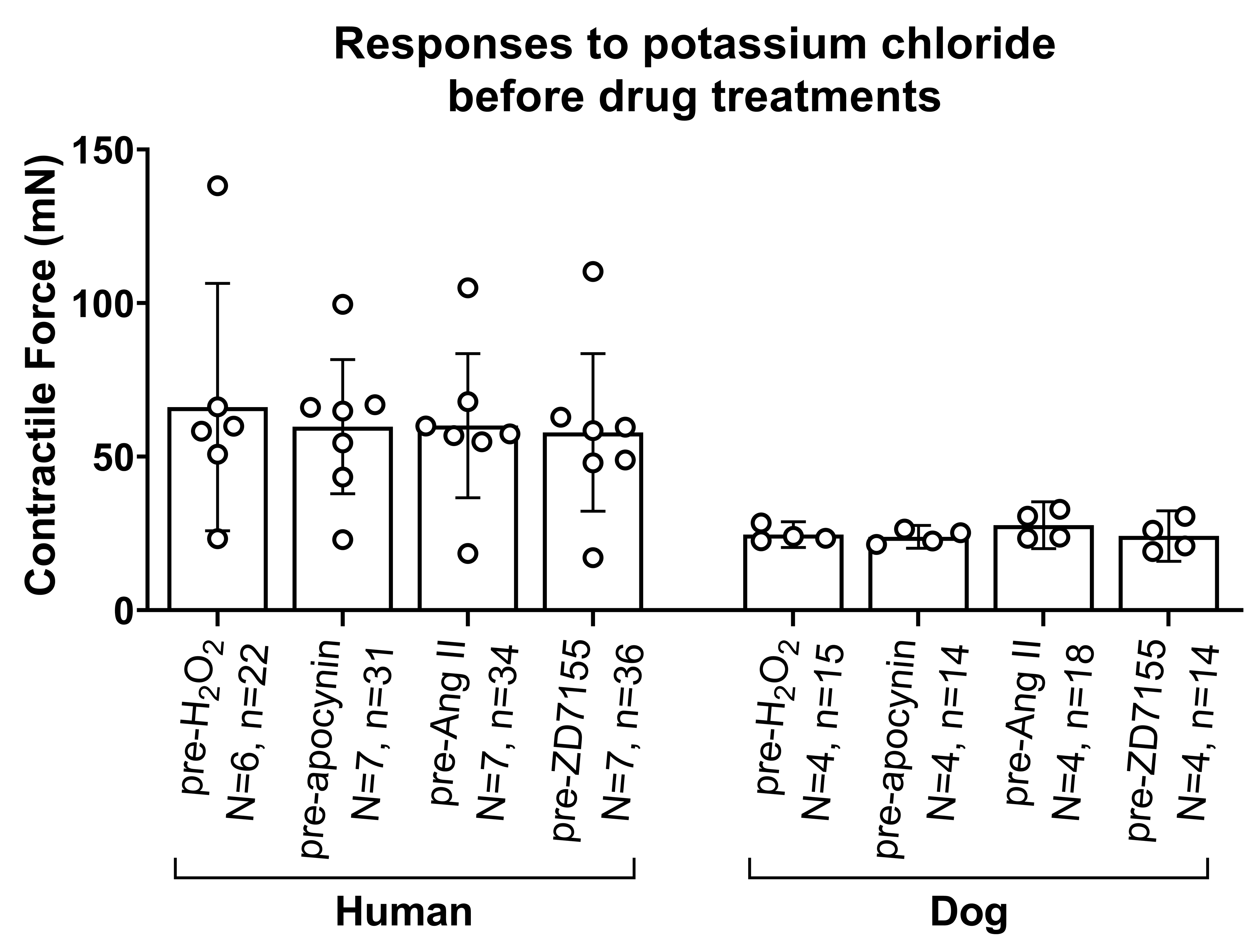

Supplement: S2 Fig — Responses to 120 mM potassium chloride (KCl) in different strips assigned to each treatment. All drug treatments applied later are indicated on the X-axis in either human or dog strips. The maximal responses to 120 mM KCl are expressed in milli Newtons (mN). N = number of bladders per group. n = number of strips per treatment. Data is presented as mean ± 95% CI. (TIF) [file pone.0287212.s002.tif]

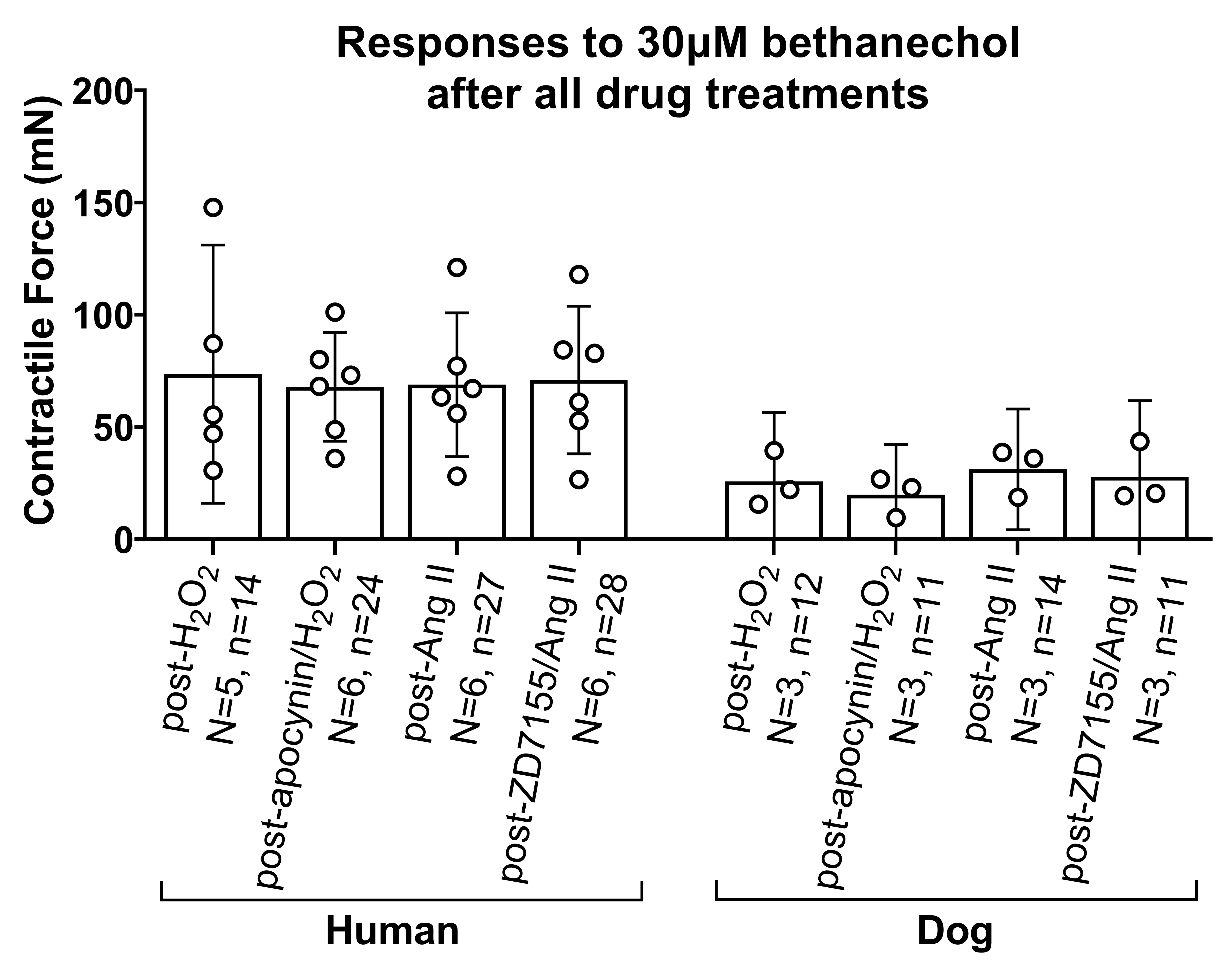

Supplement: S3 Fig — Responses to 30μM bethanechol in different strips that were already subjected to the indicated drug treatment(s). All drug treatments that were added before bethanechol treatment are indicated on the X-axis in either human or dog strips. The maximal responses to 30μM bethanechol are expressed in milli Newtons (mN). N = number of bladders per group. n = number of strips per treatment. Data is presented as mean ± 95% CI. (TIF) [file pone.0287212.s003.tif]
